# Loss of the transcription repressor ZHX3 induces senescence-associated gene expression and mitochondrial-nucleolar activation

**DOI:** 10.1371/journal.pone.0262488

**Published:** 2022-01-27

**Authors:** Tomoka Igata, Hiroshi Tanaka, Kan Etoh, Seonghyeon Hong, Naoki Tani, Tomoaki Koga, Mitsuyoshi Nakao

**Affiliations:** Department of Medical Cell Biology, Institute of Molecular Embryology and Genetics, Kumamoto University, Kumamoto, Japan; University of Massachusetts Amherst, UNITED STATES

## Abstract

Cellular senescence is accompanied by metabolic and epigenomic remodeling, but the transcriptional mechanism of this process is unclear. Our previous RNA interference-based screen of chromatin factors found that lysine methyltransferases including SETD8 and NSD2 inhibited the senescence program in cultured fibroblasts. Here, we report that loss of the zinc finger and homeobox protein 3 (ZHX3), a ubiquitously expressed transcription repressor, induced senescence-associated gene expression and mitochondrial–nucleolar activation. Chromatin immunoprecipitation–sequencing analyses of growing cells revealed that ZHX3 was enriched at the transcription start sites of senescence-associated genes such as the cyclin-dependent kinase inhibitor (*ARF-p16^INK4a^*) gene and ribosomal RNA (rRNA) coding genes. ZHX3 expression was consistently downregulated in cells with replicative or oncogene-induced senescence. Mass spectrometry-based proteomics identified 28 proteins that interacted with ZHX3, including ATP citrate lyase and RNA metabolism proteins. Loss of ZHX3 or ZHX3-interaction partners by knockdown similarly induced the expression of *p16^INK4a^* and rRNA genes. *Zhx3*-knockout mice showed upregulation of *p16^INK4a^* in the testes, thymus and skeletal muscle tissues, together with relatively short survival periods in males. These data suggested that ZHX3 plays an essential role in transcriptional control to prevent cellular senescence.

## Introduction

Cellular senescence is characterized by persistent growth arrest, senescence-associated (SA)-β-galactosidase positivity and the senescence-associated secretory phenotype, which plays important roles in tissue development, tumor suppression and aging *in vivo* [1–4]. The senescent cells also exhibit enlarged cell size and increased protein content due to the remodeling of various metabolic pathways including protein synthesis and degradation, and mitochondrial oxidative phosphorylation (OXPHOS) [1, 5]. It has been reported that many organelles such as the nucleus, nucleolus and the mitochondria undergo structural and functional changes during aging [6]. In fact, senescent cells have higher activities in ribosome biogenesis and OXPHOS than proliferating cells [7–11]. Conversely, metabolic stresses such as loss of proteostasis and mitochondrial dysfunction lead to senescent state [1]. Several lines of evidence suggest that metabolic and epigenomic remodeling cooperatively create these features of senescent cells [4], although it is still undetermined whether such reprogram is a cause or consequence of cellular senescence.

We previously performed an RNA interference (RNAi)-based screen in HeLa, HepG2 cells and IMR-90 human diploid fibroblasts, using a custom small interfering RNA (siRNA) library against 79 chromatin factors [11, 12]. Individual knockdown (KD) of these factors showed increases in either mitochondrial or nucleolar areas per cell, or both. Among the factors, two lysine methyltransferases, SETD8/PR-Set7 and NSD2/WHSC1/MMSET, are involved in the senescence-associated metabolic and epigenomic reprogramming. SETD8 methyltransferase, which catalyzes mono-methylation of histone H4 at lysine 20 (H4K20me1), inhibited nucleolar and mitochondrial activities to prevent cellular senescence, by repressing the genes encoding ribosomal proteins and ribosomal RNAs as well as the CDK inhibitor *p16^INK4A^* [11]. In contrast, NSD2 methyltransferase shapes transcriptionally active histone H3 lysine 36 trimethylation (H3K36me3) at the cell cycle-related gene loci, involving in DNA replication and cell division, to maintain proliferation and prevent cellular senescence [12]. Therefore, the loss of either SETD8 or NSD2 resulted in senescent conditions, accompanied by the secretory phenotype with metabolic activation. However, it is not well understood how transcriptional machineries are involved in the metabolic remodeling during senescence.

By screening the above-mentioned siRNA library, we identified the zinc finger and homeobox 3 (ZHX3) as an essential transcription factor involving in morphology of mitochondria and nucleolus. ZHX3 is one of the three members of the ZHX family in mammals (ZHX1, ZHX2 and ZHX3), which contains two zinc finger motifs and five homeobox DNA-binding domains, and functions as a transcriptional repressor [13, 14]. ZHX1 was first isolated as an interacting protein with the A subunit of the nuclear factor Y (NF-YA) that binds the Y-box sequence [15], and ZHX proteins form homo dimers and heterodimers with each other [16, 17]. Recent studies have revealed that the ZHX proteins are associated with cell development and differentiation, and various cancers [18–21], although their biological roles and transcriptional repressive actions remain to be elucidated.

In this study, we found that loss of the ZHX3, a ubiquitously expressed transcription repressor, induced mitochondrial–nucleolar activation and cellular senescence in human fibroblasts. Consistently, ZHX3 was downregulated in replicative and oncogene-induced senescent cells. ZHX3 was enriched at the transcription start sites of senescence-associated genes, indicating that ZHX3 is involved in transcriptional program of cellular senescence.

## Materials and methods

### Cell culture and siRNAs

IMR-90 (human diploid fibroblast, purchased from ATCC) and IMR-90 ER:Ras (H-RasG12V) cells were maintained in Dulbecco’s modified Eagle’s medium supplemented with 10% (v/v) heat-inactivated fetal bovine serum. To induce OIS, IMR-90 ER:Ras cells were treated with 100 nM 4-OHT for 6–8 days. RS cells were prepared by repeated passaging for 10–12 weeks [11]. Transfection of siRNA was performed every 3 days using RNAiMAX (Invitrogen, MA, USA). The siRNAs used in this study are listed in **S1 Table**. The siRNA libraries were previously described [12].

### Animal experiments

Animal experiments and ethics in this study were approved by The Animal Care and Use Committee in Kumamoto University, Japan. The approval numbers are A2019-098 and A2021-076. We carefully performed experiments which caused little or no discomfort to mice with short-term retention, sampling and euthanasia without awakening under anesthesia in accordance with the institutional regulation. Zhx3-KO (Zhx3^tm1.1(KOMP)Vlcg^) mice were generated by the Knockout Mouse Phenotyping Program and the International Knockout Mouse Consortium, and were purchased from the University of California, Davis. Briefly, in the KO construct, the ZEN-UB1 Velocigene cassette was inserted into *Zhx3*, replacing all coding exons and intervening sequences. Genotyping PCR was performed using appropriate primers (**S2 Table**). C57BL/6J mice were purchased from Charles River Laboratories (MA, USA). Pregnancy rates were assessed from the significant weight gains of mated females. Gene expression analyses were performed on 14 types of tissue from male mice sacrificed at 7 weeks of age.

### Reagents

Oligomycin, carbonyl cyanide 4-(trifluoromethoxy) phenylhydrazone (FCCP), rotenone, and antimycin A were used for metabolic measurement by extracellular flux analyzer (Agilent Technologies, CA, USA). Antibodies were as follows: anti-ZHX3 (ab9950) and anti-ACLY (ab40793) (Abcam, Cambridge, UK); anti-B23/NPM1 (sc-6013R, sc-271737), anti-mouse IgG (sc-2025) and anti-rabbit IgG (sc-2027) (Santa Cruz Biotechnology, Dallas, TX, USA); and anti-β-tubulin (T4026) (Sigma Aldrich, MO, USA).

### Quantitative reverse transcription PCR (RT-qPCR) analyses

Total RNA was extracted from cultured cells with TRIzol Reagent (Invitrogen), used to produce cDNA in ReverTra Ace qPCR RT Master Mix, followed by qPCR with SYBR green fluorescence using THUNDERBIRD reagent (Toyobo, Osaka, Japan) and a StepOnePlus Real-Time PCR instrument (Thermo Fisher Scientific, Waltham, MA, USA). GAPDH was used for normalization. Primer sequences are listed in **S2 Table**.

### Nuclear extraction

Nuclear extracts were prepared using an NE-PER Nuclear Cytoplasmic Extraction Reagent kit (Pierce, Rockford, IL, USA) according to the manufacturer’s instruction. Briefly, cells were washed twice with cold phosphate-buffered saline (PBS) and spun by centrifugation at 5,000 × g for 3 min. The cell pellet was resuspended in 200 μL cytoplasmic extraction reagent I by vortexing and incubated on ice for 10 min; 11 μL cytoplasmic extraction reagent II was added, vortexed for 5 sec, incubated on ice for 1 min, and spun by centrifugation at 13,500 × g for 5 min. The supernatant fraction (cytoplasmic extract) was transferred to a tube. The insoluble pellet fraction containing crude nuclei was resuspended in 100 μL nuclear extraction reagent by vortexing for 15 sec, incubated on ice for 10 min, then spun by centrifugation for 10 min at 13,500 × g. The resulting supernatant, constituting the nuclear extract, was used for the subsequent experiments.

### Western blot analysis

To prepare total cell lysates, cells were directly dissolved in sample buffer (0.1 M Tris-HCl, pH 6.8, 4% sodium dodecyl sulfate [SDS], 0.1 M DTT, 20% glycerol, 0.2% bromophenol blue). Nuclear extracts or cytoplasmic extracts were added to the same sample buffer. Proteins were separated by SDS-polyacrylamide gel electrophoresis and transferred to a Hybond ECL nitrocellulose membrane (GE Healthcare, Waukesha, WI, USA) using a semidry method. Membranes were incubated with antibodies diluted in PBS containing 5% skim milk and 0.3% Tween 20 (1:2000 for anti-ZHX3 and 1:5000 for anti-ACLY antibodies). Signals were visualized using the Western Lightning Plus-ECL (PerkinElmer, Waltham, MA, USA) and an ImageQuant LAS 4000 mini (GE Healthcare).

### Cell counting

Cells were stained with Solution 18 AO-DAPI (Chemometec, Allerod, Denmark) according to the manufacturer’s instructions and counted using an automated cell analyzer (NucleoCounter NC-250, Chemometec).

### SA-β-Gal staining

Senescence-associated-β-galactosidase (SA-β-Gal) staining was performed with the Senescence Detection Kit (BioVision, Milpitas, CA, USA), according to the manufacturer’s instruction. SA-β-Gal–positive senescent cells were identified as blue-stained cells under light microscopy. Total cells were counted using a nuclear DAPI counterstain to determine the percentage of SA-β-Gal–positive cells.

### Immunofluorescence and high-content imaging analysis

Cells were fixed with 4% paraformaldehyde for 10 min at room temperature, then permeabilized, blocked with 0.5% bovine serum albumin, and incubated with primary antibodies (each 1:500 dilution) for 1 h at room temperature, followed by incubation with Cy3- or Alexa Fluor 488-conjugated secondary antibody for 1 h. DNA was counterstained with 0.5 μg/mL DAPI.

For high-content imaging analysis, images were obtained and analyzed using a CellInsight cellomics platform with HCS studio cell analysis software (Thermo Fisher Scientific). The Spot Detector BioApplication was used to quantify the total fluorescence intensity of mitochondrial signals and to count the number of nucleoli in each cell. Each cell was defined by a DAPI channel.

RNAi screening was described previously [12]. Briefly, 2,500 IMR-90 cells were seeded with 5 nM each siRNA in 96-well plates. After 3 days, cells were fixed and subjected to immunofluorescence using an anti-mitochondrial antibody (ab3298, Abcam). Sixteen images per well were taken using CellInsight with 20× magnification. The total area of mitochondria/cell was calculated using the Spot Detector BioApplication. Screens were performed in three biological replicates and hits were defined by the magnitude of change in the mitochondrial area, with statistical analysis using Student’s t-test between control siRNA and samples.

### ChIP-qPCR and ChIP-seq analyses

ChIP assays were performed according to the Upstate Biotechnology protocol [22] with modifications. Cells were crosslinked with 1% formaldehyde for 10 min at room temperature. Twenty μL Dynabeads M-280 Sheep Anti-rabbit IgG (Thermo Fisher Scientific) were bound with 2 μg anti-ZHX3 antibodies. Nuclei were isolated and sonicated with a Picoruptor (30 times, 30 s ON/30 s OFF) (Cosmo Bio, Tokyo, Japan) to generate DNA fragments. The DNA fragments were incubated overnight at 4°C with the magnetic bead-bound antibodies. The beads were washed and de-crosslinked for 4 h at 65 °C. RNase A and Proteinase K were used for RNA and protein digestion, respectively, and the DNA was purified with a QIAquick PCR Purification Kit (Qiagen, MD, USA). For ChIP-qPCR analysis, DNA enrichment was determined with a Step One Plus system (Applied Biosystems, MA, USA), using SYBR green fluorescence. Input DNA was used to make a standard curve to determine the level of DNA enrichment. Primer sequences are listed in **S2 Table**.

For genome-wide ZHX3 distribution analysis, extracted DNA was subjected to adaptor ligation using the NEBNext Ultra II DNA Library Prep Kit for Illumina (New England Biolabs, Ipswich, MA, USA). Sequencing was performed on a NextSeq 500 (Illumina, San Diego, CA, USA) with 75-bp single-end reads, and data analyses were performed on the Galaxy platform. The reads were trimmed using Trimmomatic v.0.36.3 and mapped to the hg19 reference genome, or a custom build comprising a ribosomal DNA complete repeating unit (GenBank accession no. U13369.1), using the Burrow-Wheeler Aligner v.0.7.15.1. After removing duplicate reads using Picard MarkDuplicates v.1.136.0, the reads were normalized to those of input by deepTools bamCompare v.2.5.0.0 [23] and visualized with an Integrative Genomics Viewer. Distributions around gene loci were calculated and visualized using deepTools computeMatrix and plotProfile, respectively. The number of reads in each gene was calculated by featureCounts v.1.4.6.p5. The peak detection of ZHX3 was performed by a Model-Based Analysis of ChIP-seq (MACS v.1.0.1). ChIP-seq data for ZHX3 in IMR-90 cells were deposited in the GEO database under accession code GSE184992. ChIP-seq data for UBF, Pol IB, and input in HEK293T cells can be found on the SRA database under accession number SRP004897 [24]. Other ChIP-seq data in IMR-90 cells were obtained from the ENCODE project (https://www.encodeproject.org) [25].

### Assessment of mitochondrial activities

Real-time monitoring of cellular OCR was performed by an XF24 extracellular flux analyzer (Seahorse Bioscience, Billerica, MA, USA) as previously described [11]. The siRNA-treated cells were dissociated by trypsinization, then cultured in the assay plate for approximately 12 h before the assay. During the measurement, the following inhibitors of respiratory chain components were serially added to the culture medium: ATP synthase inhibitor, oligomycin (1 μM); respiratory uncoupler, FCCP (1.5 μM); and complex I and III inhibitors, rotenone (1 μM) and antimycin A (1 μM). OCR was measured four times.

### Immunoprecipitation

Antibodies bound to 20 μL Dynabeads M-280 sheep anti-rabbit IgG (Thermo Fisher Scientific) were subjected to covalent cross-linking. A 500-μL solution of chemical cross-linking reagent, 50 mM dimethyl pimelimidate, was freshly prepared in ice-cold 200 mM triethanolamine, pH 8.9. Non-covalently bound antibodies were desorbed by eluting with 500 μL elution buffer (0.1 M glycine-HCl, pH 2.9), and neutralized with 500 μL wash buffer (50 mM Tris-HCl, pH 8.0, 150 mM NaCl). Antibody-crosslinked beads were added to total cell lysates or nuclear extracts. The beads were then washed six times with standard low lysis buffer (50 mM Tris-HCl, pH 8.0, 5 mM EDTA, 150 mM NaCl, 0.5% NP-40, 0.5% Triton X100). Bead-bound proteins were eluted with elution buffer as described above. The immunoprecipitated proteins were used for subsequent western blot analysis or mass spectrometry analysis.

### Mass spectrometry

Immunoprecipitated proteins were separated by electrophoresis on a 5%–20% e-PAGEL (ATTO, Tokyo, Japan) and stained with a Pierce Silver Stain Kit (Thermo Fisher Scientific). For the in-gel digestion of proteins, 8–12 gel slices were excised from each lane and cut into approximately 1-mm-sized pieces. De-stained proteins in the gel pieces were reduced with 10 mM DTT (Thermo Fisher Scientific) in 25 mM ammonium bicarbonate (FUJIFILM Wako, Tokyo, Japan), alkylated with 55 mM iodoacetamide (Thermo Fisher Scientific) in 25 mM ammonium bicarbonate, and digested with trypsin and lysyl endopeptidase (Promega, WI, USA) in a buffer containing 40 mM ammonium bicarbonate, pH 8.0, overnight at 37°C. Digested peptides were then extracted with 50% acetonitrile and 0.1% formic acid (FUJIFILM Wako), and 70% acetonitrile with 0.1% formic acid. Supernatants were combined in a fresh vial and concentrated to 15 μL in a centrifugal evaporator. The concentrated samples were diluted 2-fold with 2% acetonitrile and 0.1% trifluoroacetic acid (FUJIFILM Wako). The resultant peptides were analyzed on an Advance UHPLC system (Michrom Bioresources Inc., CA, USA) coupled to a Q Exactive mass spectrometer (Thermo Fisher Scientific). Raw mass spectrum data were processed using an Xcalibur (Thermo Fisher Scientific). The raw liquid chromatography with tandem mass spectrometry data were analyzed against the SwissProt protein database, restricted to *Homo* sapiens, using Proteome Discoverer ver.1.4 (Thermo Fisher Scientific) with the Mascot search engine ver.2.4 (Matrix Science, London, UK). A decoy database comprising either randomized or reversed sequences in the target database was used for false discovery rate (FDR) estimation. Search results were filtered against a 1% FDR (**S3 Table**).

### Statistical analysis

Survival of the KO mice was estimated by the Kaplan-Meier method, and the log-rank test was used to examine differences in survival. All other data are presented as mean ± standard deviation, and were statistically analyzed by a two-tailed Student’s t-test.

## Results and discussion

### Loss of ZHX3 induces cellular senescence

**Fig 1:**
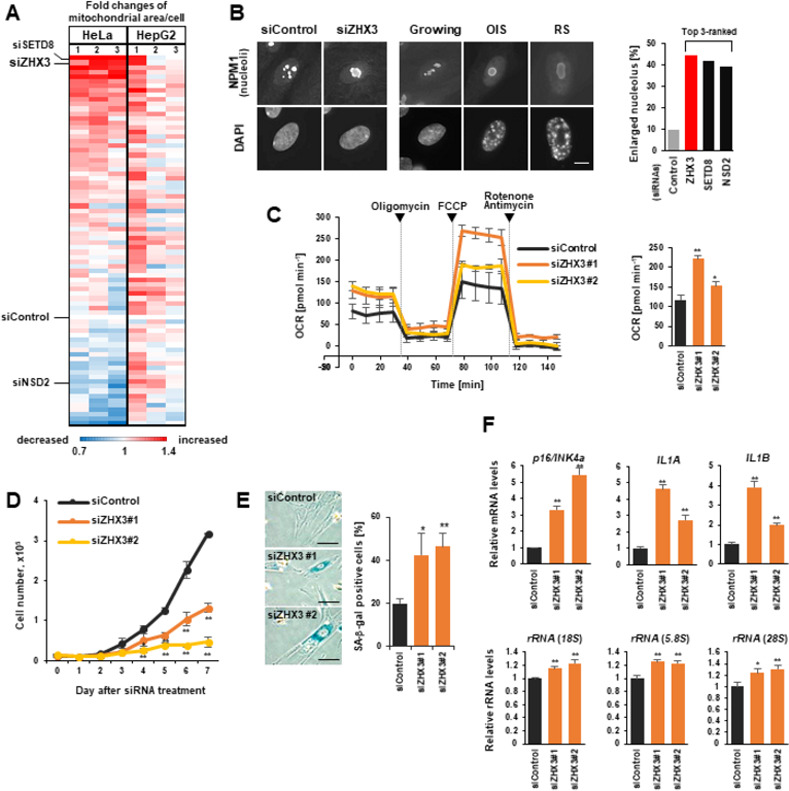
Loss of ZHX3 induces cellular senescence. **(A)** Heat map of mitochondrial area/cell in a 79 siRNA-based knockdown (KD) in HeLa and HepG2 cells. Fold changes relative to control KD (siControl) are shown. Positions of ZHX3, SETD8 and NSD2 are indicated. Each KD was repeated three times (n>1,200 cells) [12]. **(B)** Representative immunofluorescent images of nucleoli in ZHX3-KD (day 3), growing and senescent (OIS and RS) IMR-90 fibroblasts. Nucleoli and DNA were detected using nucleophosmin (NPM1) and DAPI, respectively. Scale bar, 10 μm. The proportion containing one or two enlarged nucleoli/cell (n>100 cells each) was measured using the Spot Detector BioApplication (right). **(C)** OCR in control and ZHX3-KD IMR-90 cells (day 6). Respiratory chain inhibitors were serially added to the medium at the indicated time points. Respiratory capacities were calculated by subtracting the rotenone/antimycin-treated value from the FCCP-treated value (right). **(D)** Growth curves in control and ZHX3-KD IMR-90 cells; siRNA transfections were performed on days 0 and 4. **(E)** SA-β-galactosidase (SA-β-gal) staining of control and ZHX3-KD IMR-90 cells (day 6, each n > 100 cells). Scale bars, 100 μm. **(F)** RT-qPCR analysis of senescence-related genes *p16*^*INK4a*^, *IL1A/B* and ribosomal RNAs in control and ZHX3-KD IMR-90 cells (day 3). Values shown are mean +/- standard deviation from three independent experiments, using the Student’s t-test (*p<0.05, **p<0.01).

In this study, we found that depletion of the transcriptional repressor ZHX3 [16, 17] using three independent siRNAs (**S1A and S1B Fig**) significantly augmented both mitochondrial and nucleolar areas per cell, compared with control KD (**Figs 1A and 1B and S1C**). Like the loss of SETD8 or NSD2, ZHX3 KD led to an increased mitochondrial oxygen consumption rate (OCR) in IMR90 cells (**Fig 1C**), resulting in mitochondrial activation. Further, ZHX3-KD cells exhibited growth inhibition and senescence-associated (SA)-β-galactosidase positivity on day 6 after siRNA transfection (**Fig 1D and 1E**). To test the involvement of ZHX3 in senescence-related gene expression, we performed reverse transcription-quantitative PCR (RT-qPCR) analyses. Levels of transcription for cyclin-dependent kinase inhibitor (*p16^INK4a^*), inflammatory cytokines (*IL-1A/1B*) and nucleolus-related ribosomal RNA (rRNA) genes were upregulated in ZHX3-KD cells (**Fig 1F**; *p14^ARF^* results shown in **S1D Fig**). There are three ZHX family proteins, but only the loss of ZHX3, not ZHX1 or ZHX2, derepressed *p16^INK4a^* (**S1E Fig**), suggesting a unique function of ZHX3 in cellular senescence.

### ZHX3 as a repressor is enriched at senescence-associated target genes

**Fig 2:**
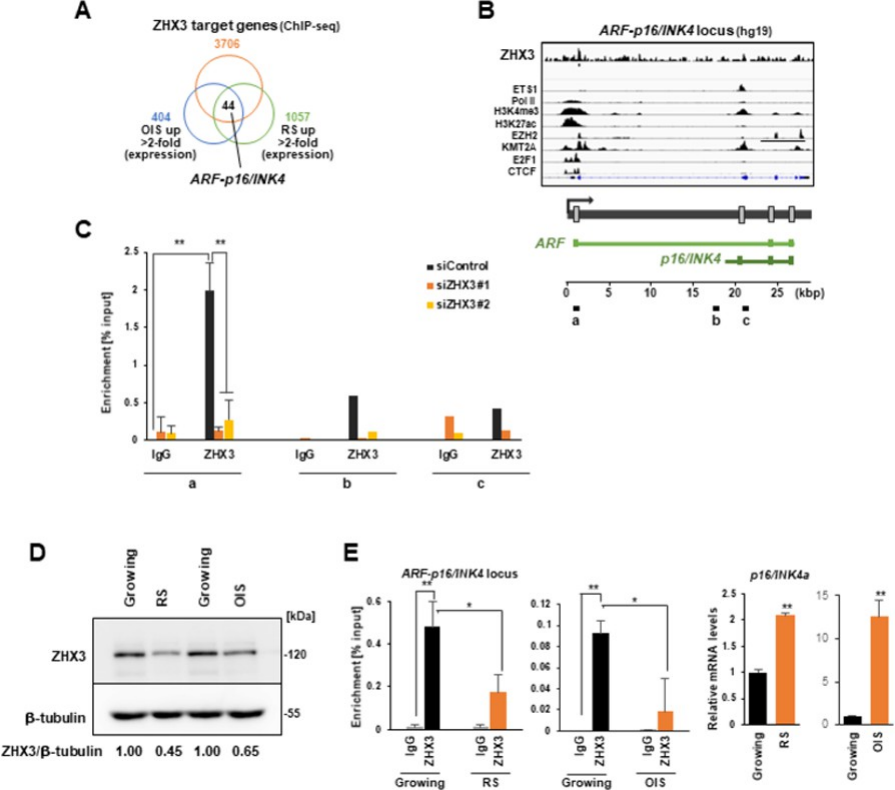
ZHX3 as a repressor is enriched at senescence-associated target genes. **(A)** Venn diagram showing overlap between ZHX3-enriched genes identified by ChIP-seq analysis and commonly upregulated genes in OIS and RS IMR-90 cells. Transcriptome data of OIS and RS cells were obtained from the Gene Expression Omnibus (GEO, accession no. GSE86546). **(B)** Integrative Genomics Viewer tracks showing the distribution of ZHX3 in the *ARF-p16*^*INK4a*^ gene locus. Data on ETS1, RNA Pol II, H3K4me3, H3K27ac, EZH2, KMT2A, E2F1 and CTCF were obtained from ENCODE datasets. The amplification sites in ChIP-qPCR are shown (a, b and c). **(C)** ChIP-qPCR analysis of ZHX3 in the *ARF-p16*^*INK4a*^ locus in control and ZHX3-KD cells (day 3). Values shown are mean +/- standard deviation from three independent experiments, using the Student’s t-test (*p<0.05, **p<0.01). **(D)** Western blot analysis of ZHX3 in growing, RS and OIS IMR-90 cells. Ratios for ZHX3/β-tubulin bands are assessed with densitometry. **(E)** ChIP-qPCR analysis of ZHX3 in the *ARF-p16*^*INK4a*^ locus (site a) in growing, RS and OIS cells, together with RT-qPCR analysis of *p16*^*INK4a*^ expression.

To clarify the target genes of ZHX3, we performed chromatin immunoprecipitation–sequencing (ChIP-seq) analyses of proliferating IMR-90 cells using antibodies against ZHX3. ZHX3 was remarkably enriched at the transcription start site (TSS) of each target gene (**S2A Fig**). Combined with our transcriptome data from growing, oncogene-induced senescence (OIS) and replicative senescence (RS) IMR-90 cells [10], we have found 44 upregulated genes (> 2-fold) with enrichment of ZHX3 in senescent cells (**Fig 2A**; results for 51 downregulated genes shown in **S2B Fig**). Gene set enrichment analysis revealed ‘cell division’ as the top-ranked gene set among the upregulated genes (**S2C Fig**), which included *ARF*-*p16^INK4a^* [26, 27]. Indeed, ZHX3 was significantly enriched at the TSS of *ARF*-*p16^INK4a^* (**Fig 2B**; rRNA genes [24] shown in **S2D Fig**). Using ChIP-qPCR, we confirmed that ZHX3 enrichment, especially at TSS site a, was decreased in ZHX3-KD cells (**Figs 2C and S2D**). Our data suggested that ZHX3 may protect cellular senescence by repressing senescence-related genes such as *ARF*-*p16^INK4a^*. We further found that ZHX3 protein expression was downregulated in RS and OIS cells compared with growing cells (**Fig 2D**). Consistently, ChIP-qPCR and RT-qPCR analyses in senescent cells showed that ZHX3 enrichment at *ARF*-*p16^INK4a^* was decreased, while *p16^INK4a^* mRNA expression was induced (**Fig 2E**; rRNA genes shown in **S3A Fig**), suggesting that ZHX3 downregulation promotes cellular senescence.

### ZHX3 cooperates with RNA metabolism proteins

**Fig 3:**
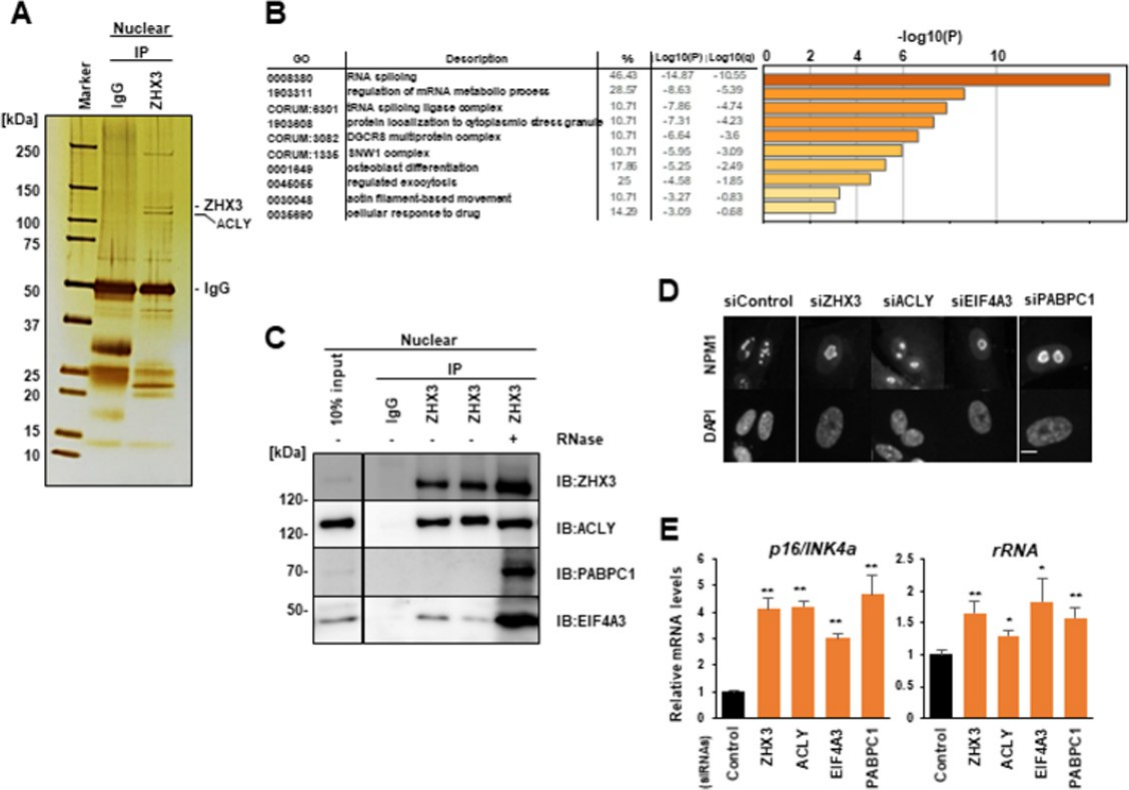
ZHX3 cooperates with RNA metabolism proteins. **(A)** Silver staining of proteins immunoprecipitated from nuclear extracts of proliferating IMR-90 cells using IgG and anti-ZHX3 antibodies. Mass spectrometry-based proteomics analyses identified 28 proteins that interacted with ZHX3; estimated bands for ZHX3 and ATP citrate lyase (ACLY) are indicated. **(B)** Gene Ontology analysis of the ZHX3-interacting proteins, which mostly comprised RNA-related proteins. **(C)** Western blot analysis of proteins immunoprecipitated using IgG and anti-ZHX3 antibodies from IMR-90 nuclear extracts, with (+) or without (-) RNase treatment. Antibodies against ZHX3, ACLY, PABPC1 and EIF4A3 were used for detection. **(D)** Representative immunofluorescent images of enlarged nucleoli in IMR-90 cells (day 6) with ZHX3-, ACLY-, EIF4A3-, or PABPC1-KD. DNA was counterstained with DAPI. Scale bar, 10 μm. Quantitative data of the cells that had enlarged nucleoli are shown in **S3C Fig**. **(E)** RT-qPCR analysis of *p16*^*INK4a*^ and ribosomal RNA genes on day 6 of each KD in IMR-90 cells. Values shown are mean +/- standard deviation from three independent experiments, using the Student’s t-test (*p<0.05, **p<0.01).

To investigate the mechanism of transcriptional control by ZHX3, we used a mass spectrometry-based proteomics approach. Of the 28 proteins that were found to interact with ZHX3 (**Fig 3A and S3 Table**), Gene Ontology analysis from repeated experiments identified ZHX3 partners that included ATP citrate lyase (ACLY) and the RNA metabolism proteins, poly(A)-binding protein 1 (PABPC1) and eukaryotic translation initiation factor 4A-3 (EIF4A3) [28, 29] (**Fig 3B**). Next we confirmed that ACLY co-immunoprecipitated with ZHX3, and that PABPC1 and EIF4A3 bound efficiently to ZHX3 in the presence of RNase treatment to disturb RNA–protein interactions (**Fig 3C**). Interestingly, loss of any one of these proteins, or ZHX3 itself (**S3B Fig**), induced significant nucleolar enlargement (**Fig 3D**) and upregulated transcription of *p16^INK4a^* and rRNA (**Fig 3E**), suggesting that ZHX3 and its partners interact to cooperatively protect cellular senescence.

### ZHX3 is involved in senescent phenotypes

**Fig 4:**
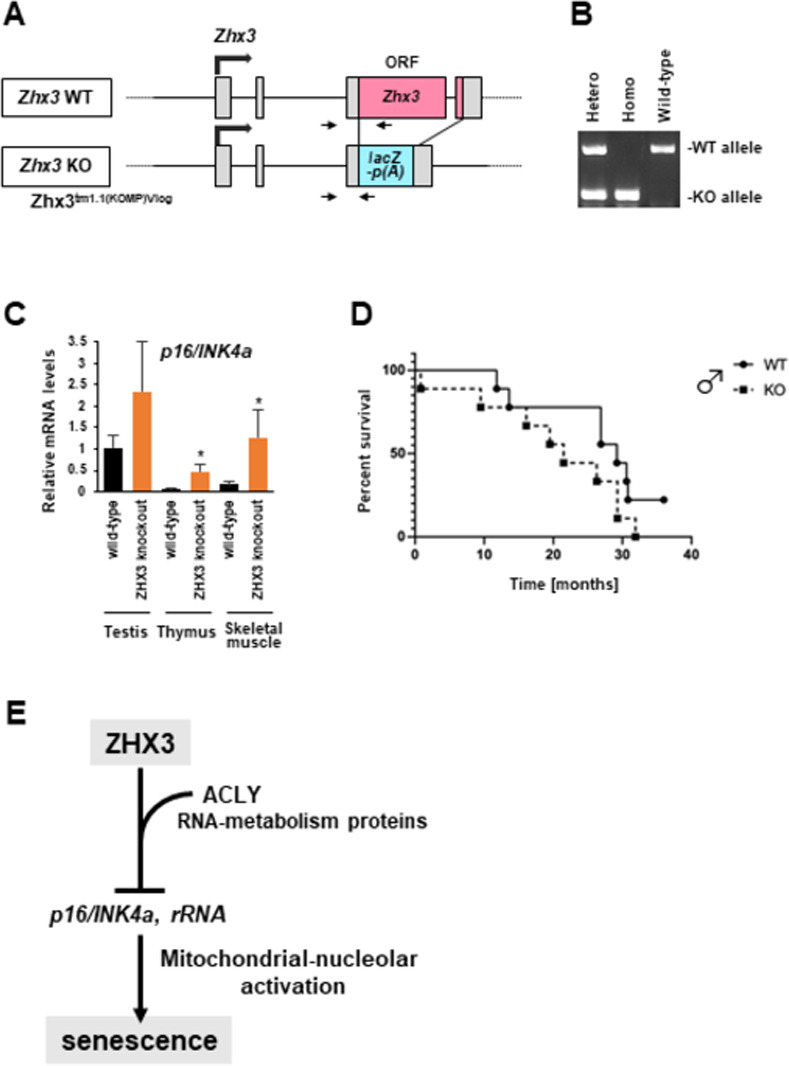
ZHX3 is involved in senescent phenotypes. **(A)** Schematic diagram of the *Zhx3* gene locus in wild type (WT) and Zhx3-knockout (KO) alleles. Arrows indicate primers used for genotyping PCR. **(B)** Genotyping PCR of WT, heterozygous and homozygous Zhx3-KO mice. **(C)** RT-qPCR analysis of *p16*^*INK4a*^ in tissues of 7-week-old WT and KO mice. Values shown are mean +/- standard deviation (each n = 3), using the Student’s t-test (*p<0.05, **p<0.01). **(D)** Kaplan-Meier survival curves of male WT and Zhx3-KO mice (each n = 9). **(E)** Proposed model of the role of ZHX3 in preventing cellular senescence. ZHX3 and its cofactors (ACLY and RNA metabolism proteins) suppress *p16*^*INK4a*^ and ribosomal RNA gene transcription, which promotes mitochondrial–nucleolar activation in senescent cells.

Finally, to clarify the role of Zhx3 *in vivo*, we prepared a *Zhx3* knockout (KO) mouse using the Zhx3^tm1.1(KOMP)Vlcg^ allele, which replaces the open reading frame with *lacZ-p(A)* (**Fig 4A and 4B**). *Zhx3* KO mice showed no apparent progeroid-like phenotypes such as hair loss or graying and bone abnormalities (data not shown). We examined 14 tissues from wild-type and KO mice (**S4A Fig**), and found that *Zhx3* gene was highly expressed in some tissues such as the testis of wild-type mice. Among the tissues studied, *p16^INK4a^* expression tended to be upregulated in the testes, thymus and skeletal muscle of *Zhx3*-KO mice (**Fig 4C**). Additionally, we observed that the ratio of homozygous *Zhx3*-KO pups from heterozygous intercrosses was lower than expected, probably because of gestational or perinatal problems (**S4B and S4C Fig**). Although we did not conclude gender differences due to the limited numbers of *Zhx3*-KO mice used, *Zhx3*-KO males appeared to have relatively short survival periods (**Fig 4D**; KO females shown in **S4D Fig**). Phenotyping by the International Mouse Phenotyping Consortium (www.mousephenotype.org) showed that *Zhx3*-KO mice had significantly decreased grip strength and auditory brain stem response, and increased bone mineral density and lean body mass.

Collectively, we propose a model (**Fig 4E**) whereby ZHX3 and its interacting proteins repress senescence-related genes (*p16^INK4a^* and rRNAs) and mitochondrial–nucleolar activities in growing cells. Thus, loss of ZHX3 transcriptionally induces senescence-related genes and therefore causes p16^INK4a^/RB-mediated metabolic activation [10, 11], which lead to cellular senescence. Although we successfully identified ZHX3-interacting proteins, further studies are necessary to understand how ZHX3 complex represses transcription of senescence-associated genes. To date, it has been reported that attenuated ZHX3 expression is correlated with progression of several tumors such as renal cell carcinoma, breast cancer and bladder carcinoma [19–21], indicating that ZHX3 is involved in both senescent and oncogenic programs.

## Conclusions

RNA interference-based screening revealed that loss of the transcription repressor ZHX3 induced cellular senescence in human fibroblasts. Indeed, ZHX3 was downregulated in replicative and oncogene-induced senescent cells. Epigenomics and proteomics analyses revealed that ZHX3 was enriched at the transcription start sites of *ARF-p16^INK4a^* and ribosomal RNA genes, and that loss of ZHX3 or its cofactors derepressed gene expression and led to mitochondrial–nucleolar activation.

## Supporting information

S1 Fig Analysis of ZHX3-knockdown cells. **(A)** RT-qPCR analysis of *ZHX3* expression on day 3 in IMR-90 cells undergoing ZHX3-KD compared with Control-KD. **(B)** Western blot analysis of ZHX3 on day 1 and day 2 of Control- or ZHX3-KD in IMR-90 cells. **(C)** Proportions of IMR-90 cells containing one or two nucleoli in Control- and ZHX3-KD. The number of nucleoli/cell was calculated by measuring the number of fluorescence signals of nucleophosmin (B23) in a single cell (each n >1,000 cells). **(D)** RT-qPCR analysis of *p14/ARF* on day 3 of ZHX3-KD compared with Control-KD in IMR-90 cells. **(E)** RT-qPCR analysis of *p16*^*INK4a*^ on day 3 of ZHX1-, ZHX 2- or ZHX 3-KD compared with Control-KD in IMR-90 cells. Values shown are the mean +/- standard deviation from three independent experiments, using the Student’s t-test (*p<0.05, **p<0.01).
(TIF)

S2 Fig ChIP-seq analysis of ZHX3. **(A)** ChIP-seq analysis showing the distribution of ZHX3 around target gene loci in proliferating IMR-90 cells. TSS, transcription start site; TES, transcription end site. **(B)** Venn diagram showing overlap between ZHX3-enriched genes and genes commonly downregulated in IMR-90 cells undergoing OIS or RS. Transcriptome data of OIS and RS cells were obtained from GSE86546. **(C)** Gene Ontology analyses of 44 and 51 ZHX3-enriched genes that were upregulated and downregulated, respectively, in both OIS and RS IMR-90 cells. **(D)** Integrative Genomics Viewer tracks showing the distribution of ZHX3 in a ribosomal DNA complete repeating unit (U13369.1). Data for UBF and RNA Pol IB were obtained from the SRA database under the accession number SRP004897. Bars indicate the PCR amplification sites. ChIP-qPCR analysis of ZHX3 at ribosomal DNA loci in Control- or ZHX3-KD cells (day 3). Values shown are the mean +/- standard deviation from three independent experiments, using the Student’s t-test (*p<0.05, **p<0.01).
(TIF)

S3 Fig Analysis of ZHX3 function. **(A)** ChIP-qPCR analysis of ZHX3 at ribosomal DNA loci, and RT-qPCR analyses of ribosomal RNA in growing, RS and OIS cells. For RS, IMR-90 cells were repeatedly passaged for 10 weeks. OIS was induced by expressing oncogenic Ras (H-rasV12) for 6 days. **(B)** RT-qPCR analysis of individual KD of *ZHX3*, *ACLY*, *EIF4A3* and *PABPC1* compared with Control-KD in IMR-90 cells (day 3). Values shown are the mean +/- standard deviation from three independent experiments, using the Student’s t-test (*p<0.05, **p<0.01). **(C)** Quantitative data of the cells that had enlarged nucleoli are shown (each >20 cells).
(TIF)

S4 Fig Analyses of *Zhx3*-KO mice. **(A)** RT-qPCR analyses of *Zhx3* in wild-type and *Zhx3*-KO mice. Technical replicates of the same sample were performed (each n = 3). **(B)** Genotype ratios of heterozygous intercrosses in male and female pups (n = 29 males and 21 females). **(C)** Pregnancy rates of male and female *Zhx3*-KO mice. Total counts are 128 for mating and 88 for pregnancy. **(D)** Survival curve of female *Zhx3*-KO mice (each n = 9).
(TIF)

S1 Raw images Original uncropped and unadjusted images. (TIF)

S1 Table Sequences of siRNAs used in gene knockdown. (TIF)

S2 Table Sequences of primers used for PCR amplification. (TIF)

S3 Table Identification of proteins that interact with ZHX3 using mass spectrometry-based proteomics analysis. Cellular proteins were immunoprecipitated with anti-ZHX3 antibodies, followed by liquid chromatography in tandem with mass spectrometry analyses. The number of Mascot scores, peptide hits and peptide-spectrum matches (PSMs) are included in the list. Non-specific proteins are shaded in gray.
(TIF)

## References

[1] Wiley CD, Campisi J. From Ancient pathways to aging cells-connecting metabolism and cellular senescence. Cell Metab. 2016; 23(6): 1013–1021. doi: 10.1016/j.cmet.2016.05.010 27304503 PMC4911819

[2] Booth LN, Brunet A. The aging epigenome. Mol Cell. 2016; 62(5): 728–744. doi: 10.1016/j.molcel.2016.05.013 27259204 PMC4917370

[3] Gorgoulis V, Adams PD, Alimonti A, Bennett DC, Bischof O, Bishop C, et al. Cellular senescence: defining a path forward. Cell. 2019; 179(4): 813–827. doi: 10.1016/j.cell.2019.10.005 31675495

[4] Nakao M, Tanaka H, Koga T. Cellular senescence variation by metabolic and epigenomic remodeling. Trends Cell Biol. 2020; 30(12): 919–922. doi: 10.1016/j.tcb.2020.08.009 32978041

[5] Salama R, Sadaie M, Hoare M, Narita M. Cellular senescence and its effector programs. Genes Dev. 2014; 28 (2): 99–114. doi: 10.1101/gad.235184.113 24449267 PMC3909793

[6] Bouska M, Huang K, Kang P, Bai H. Organelle aging: Lessons from model organisms. J Genet Genomics. 2019; 46 (4): 171–185. doi: 10.1016/j.jgg.2019.03.011 31080045 PMC6553499

[7] Hutter E, Renner K, Pfister G, Stockl P, Jansen-Durr P, Gnaiger E. Senescence-associated changes in respiration and oxidative phosphorylation in primary human fibroblasts. Biochem J. 2004; 380 (Pt 3): 919–928. doi: 10.1042/BJ20040095 15018610 PMC1224220

[8] Quijano C, Cao L, Fergusson MM, Romero H, Liu J, Gutkind S, et al. Oncogene-induced senescence results in marked metabolic and bioenergetic alterations. Cell Cycle. 2012; 11 (7): 1383–1392. doi: 10.4161/cc.19800 22421146 PMC3350879

[9] Kaplon J, Zheng L, Meissl K, Chaneton B, Selivanov VA, Mackay G, et al. A key role for mitochondrial gatekeeper pyruvate dehydrogenase in oncogene-induced senescence. Nature. 2013; 498 (7452): 109–112. doi: 10.1038/nature12154 23685455

[10] Takebayashi S, Tanaka H, Hino S, Nakatsu Y, Igata T, Sakamoto A, et al. Retinoblastoma protein promotes oxidative phosphorylation through up-regulation of glycolytic genes in oncogene-induced senescent cells. Aging Cell. 2015; 14(4): 689–697. doi: 10.1111/acel.12351 26009982 PMC4531082

[11] Tanaka H, Takebayashi SI, Sakamoto A, Igata T, Nakatsu Y, Saitoh N, et al. The SETD8/PR-Set7 methyltransferase functions as a barrier to prevent senescence-associated metabolic remodeling. Cell Rep. 2017; 18 (9): 2148–2161. doi: 10.1016/j.celrep.2017.02.021 28249161

[12] Tanaka H, Igata T, Etoh K, Koga T, Takebayashi SI, Nakao M. The NSD2/WHSC1/MMSET methyltransferase prevents cellular senescence-associated epigenomic remodeling. Aging Cell. 2020; 19(7): e13173. doi: 10.1111/acel.13173 32573059 PMC7433007

[13] Barthelemy I, Carramolino L, Gutiérrez J, Barbero JL, Márquez G, Zaballos A. zhx-1: a novel mouse homeodomain protein containing two zinc-fingers and five homeodomains. Biochem Biophys Res Commun. 1996; 224(3): 870–876. doi: 10.1006/bbrc.1996.1114 8713137

[14] Liu Y, Ma D, Ji C. Zinc fingers and homeoboxes family in human diseases. Cancer Gene Ther. 2015; 22(5): 223–226. doi: 10.1038/cgt.2015.16 25857360

[15] Hirano S, Yamada K, Kawata H, Shou Z, Mizutani T, Yazawa T, et al. Rat zinc-fingers and homeoboxes 1 (ZHX1), a nuclear factor-YA-interacting nuclear protein, forms a homodimer. Gene. 2002; 290(1-2): 107–114. doi: 10.1016/s0378-1119(02)00553-x 12062805

[16] Yamada K, Kawata H, Shou Z, Hirano S, Mizutani T, Yazawa T, et al. Analysis of zinc-fingers and homeoboxes (ZHX)-1-interacting proteins: molecular cloning and characterization of a member of the ZHX family, ZHX3. Biochem J. 2003; 373(Pt 1): 167–178. doi: 10.1042/BJ20021866 12659632 PMC1223464

[17] Kawata H, Yamada K, Shou Z, Mizutani T, Miyamoto K. The mouse zinc-fingers and homeoboxes (ZHX) family; ZHX2 forms a heterodimer with ZHX3. Gene. 2003; 323: 133–140. doi: 10.1016/j.gene.2003.09.013 14659886

[18] Suehiro F, Nishimura M, Kawamoto T, Kanawa M, Yoshizawa Y, Murata H, et al. Impact of zinc fingers and homeoboxes 3 on the regulation of mesenchymal stem cell osteogenic differentiation. Stem Cells Dev. 2011; 20 (9): 1539–1547. doi: 10.1089/scd.2010.0279 21174497

[19] Kwon RJ, Kim YH, Jeong DC, Han ME, Kim JY, Liu L, et al. Expression and prognostic significance of zinc fingers and homeoboxes family members in renal cell carcinoma. PLoS One. 2017; 12(2): e0171036. doi: 10.1371/journal.pone.0171036 28152006 PMC5289508

[20] You Y, Ma Y, Wang Q, Ye Z, Deng Y, Bai F. Attenuated ZHX3 expression serves as a potential biomarker that predicts poor clinical outcomes in breast cancer patients. Cancer Manag Res. 2019; 11: 1199–1210. doi: 10.2147/CMAR.S184340 30787639 PMC6368119

[21] Deng M, Wei W, Duan J, Chen R, Wang N, He L, et al. ZHX3 promotes the progression of urothelial carcinoma of the bladder via repressing of RGS2 and is a novel substrate of TRIM21. Cancer Sci. 2021; 112(5): 1758–1771. doi: 10.1111/cas.14810 33440047 PMC8088937

[22] Dacwag CS, Ohkawa Y, Pal S, Sif S, Imbalzano AN. The protein arginine methyltransferase Prmt5 is required for myogenesis because it facilitates ATP-dependent chromatin remodeling. Mol Cell Biol. 2007; 27: 384–394. doi: 10.1128/MCB.01528-06 17043109 PMC1800640

[23] Ramírez F, Ryan DP, Grüning B, Bhardwaj V, Kilpert F, Richter AS, et al. deepTools2: a next generation web server for deep-sequencing data analysis. Nucleic Acids Res. 2016; 44: W160–W165. doi: 10.1093/nar/gkw257 27079975 PMC4987876

[24] Zentner GE, Saiakhova A, Manaenkov P, Adams MD, Scacheri PC. Integrative genomic analysis of human ribosomal DNA. Nucleic Acids Res. 2011; 39: 4949–4960. doi: 10.1093/nar/gkq1326 21355038 PMC3130253

[25] ENCODE Project Consortium. An integrated encyclopedia of DNA elements in the human genome. Nature. 2012; 489: 57–74. doi: 10.1038/nature11247 22955616 PMC3439153

[26] Gil J, Peters G. Regulation of the INK4b-ARF-INK4a tumour suppressor locus: all for one or one for all. Nat Rev Mol Cell Biol. 2006; 7(9): 667–677. doi: 10.1038/nrm1987 16921403

[27] Hirosue A, Ishihara K, Tokunaga K, Watanabe T, Saitoh N, Nakamoto M, et al. Quantitative assessment of higher-order chromatin structure of the INK4/ARF locus in human senescent cells. Aging Cell. 2012; 11(3): 553–556. doi: 10.1111/j.1474-9726.2012.00809.x 22340434

[28] Keene JD. Ribonucleoprotein infrastructure regulating the flow of genetic information between the genome and the proteome. Proc Natl Acad Sci USA. 2001; 98(13): 7018–7024. doi: 10.1073/pnas.111145598 11416181 PMC34616

[29] Wellen KE, Hatzivassiliou G, Sachdeva UM, Bui TV, Cross JR, Thompson CB. ATP-citrate lyase links cellular metabolism to histone acetylation. Science. 2009; 324 (5930): 1076–1080. doi: 10.1126/science.1164097 19461003 PMC2746744

