# Supplementary figures and images for: Loss of the transcription repressor ZHX3 induces senescence-associated gene expression and mitochondrial-nucleolar activation
Source: PLoS One. 2022 Jan 27;17(1):e0262488. doi: 10.1371/journal.pone.0262488 (PMC8794122; doi:10.1371/journal.pone.0262488)

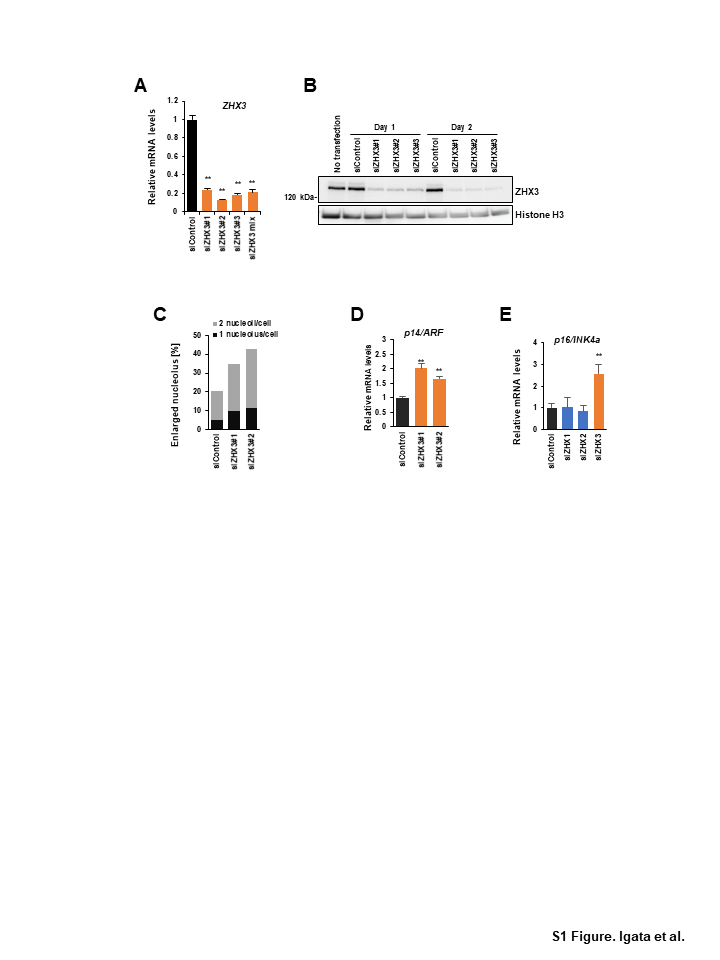

Supplement: S1 Fig — (A) RT-qPCR analysis of ZHX3 expression on day 3 in IMR-90 cells undergoing ZHX3-KD compared with Control-KD. (B) Western blot analysis of ZHX3 on day 1 and day 2 of Control- or ZHX3-KD in IMR-90 cells. (C) Proportions of IMR-90 cells containing one or two nucleoli in Control- and ZHX3-KD. The number of nucleoli/cell was calculated by measuring the number of fluorescence signals of nucleophosmin (B23) in a single cell (each n >1,000 cells). (D) RT-qPCR analysis of p14/ARF on day 3 of ZHX3-KD compared with Control-KD in IMR-90 cells. (E) RT-qPCR analysis of p16INK4a on day 3 of ZHX1-, ZHX 2- or ZHX 3-KD compared with Control-KD in IMR-90 cells. Values shown are the mean +/- standard deviation from three independent experiments, using the Student’s t-test (*p<0.05, **p<0.01). (TIF) [file pone.0262488.s001.TIF]

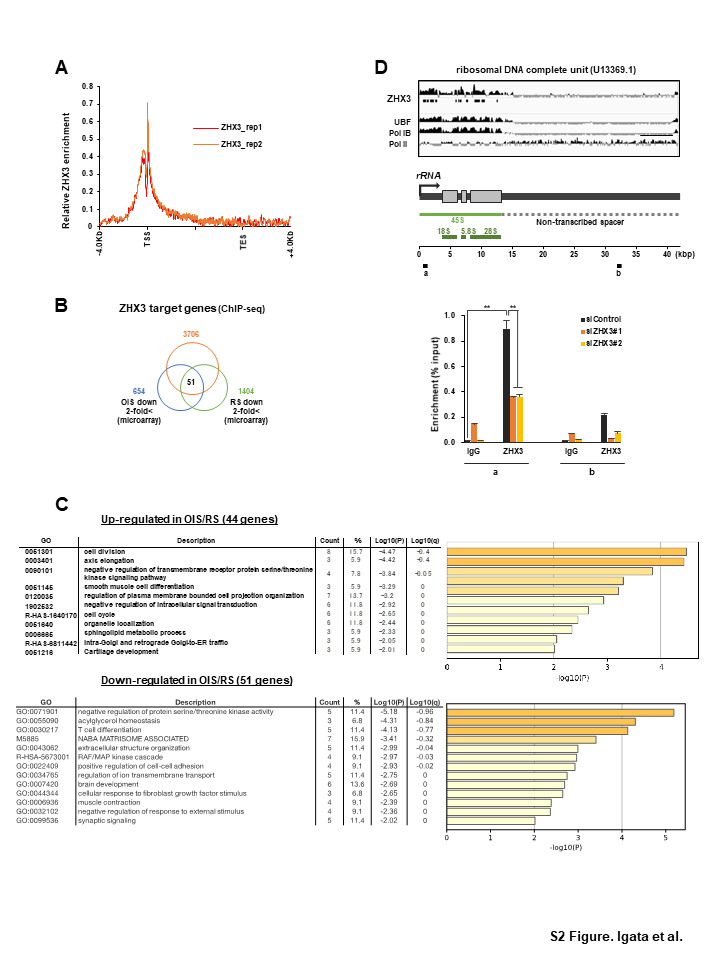

Supplement: S2 Fig — (A) ChIP-seq analysis showing the distribution of ZHX3 around target gene loci in proliferating IMR-90 cells. TSS, transcription start site; TES, transcription end site. (B) Venn diagram showing overlap between ZHX3-enriched genes and genes commonly downregulated in IMR-90 cells undergoing OIS or RS. Transcriptome data of OIS and RS cells were obtained from GSE86546. (C) Gene Ontology analyses of 44 and 51 ZHX3-enriched genes that were upregulated and downregulated, respectively, in both OIS and RS IMR-90 cells. (D) Integrative Genomics Viewer tracks showing the distribution of ZHX3 in a ribosomal DNA complete repeating unit (U13369.1). Data for UBF and RNA Pol IB were obtained from the SRA database under the accession number SRP004897. Bars indicate the PCR amplification sites. ChIP-qPCR analysis of ZHX3 at ribosomal DNA loci in Control- or ZHX3-KD cells (day 3). Values shown are the mean +/- standard deviation from three independent experiments, using the Student’s t-test (*p<0.05, **p<0.01). (TIF) [file pone.0262488.s002.TIF]

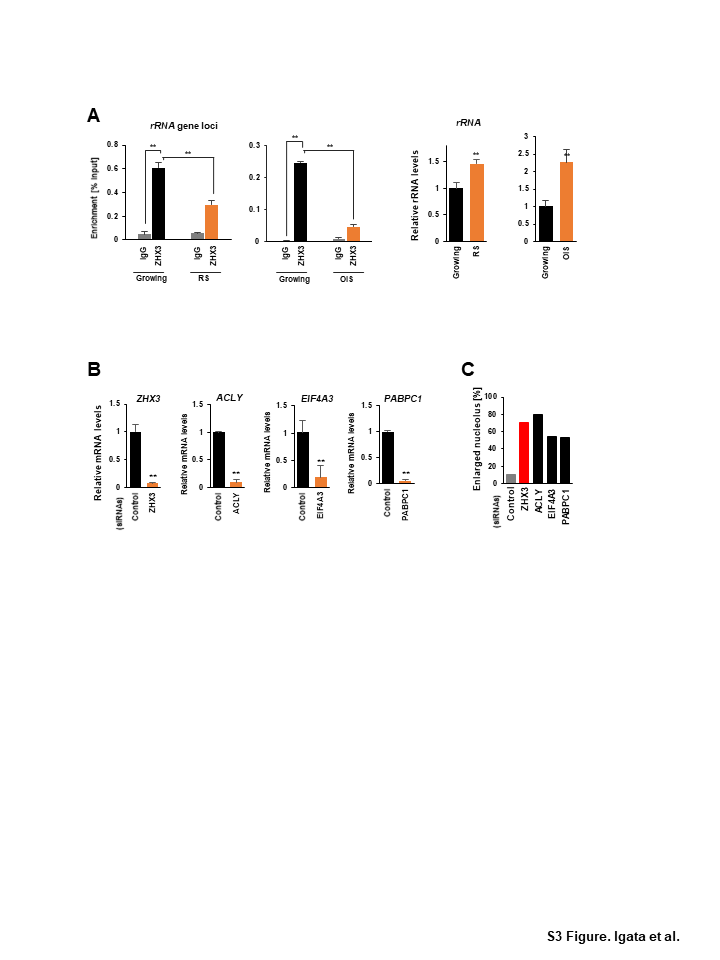

Supplement: S3 Fig — (A) ChIP-qPCR analysis of ZHX3 at ribosomal DNA loci, and RT-qPCR analyses of ribosomal RNA in growing, RS and OIS cells. For RS, IMR-90 cells were repeatedly passaged for 10 weeks. OIS was induced by expressing oncogenic Ras (H-rasV12) for 6 days. (B) RT-qPCR analysis of individual KD of ZHX3, ACLY, EIF4A3 and PABPC1 compared with Control-KD in IMR-90 cells (day 3). Values shown are the mean +/- standard deviation from three independent experiments, using the Student’s t-test (*p<0.05, **p<0.01). (C) Quantitative data of the cells that had enlarged nucleoli are shown (each >20 cells). (TIF) [file pone.0262488.s003.TIF]

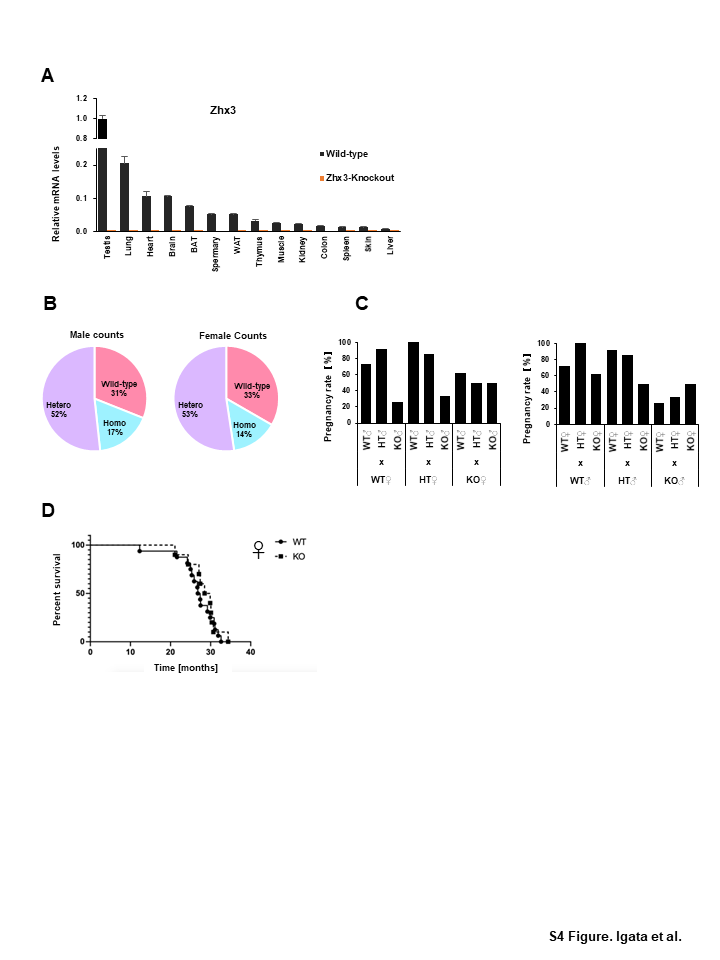

Supplement: S4 Fig — (A) RT-qPCR analyses of Zhx3 in wild-type and Zhx3-KO mice. Technical replicates of the same sample were performed (each n = 3). (B) Genotype ratios of heterozygous intercrosses in male and female pups (n = 29 males and 21 females). (C) Pregnancy rates of male and female Zhx3-KO mice. Total counts are 128 for mating and 88 for pregnancy. (D) Survival curve of female Zhx3-KO mice (each n = 9). (TIF) [file pone.0262488.s004.TIF]

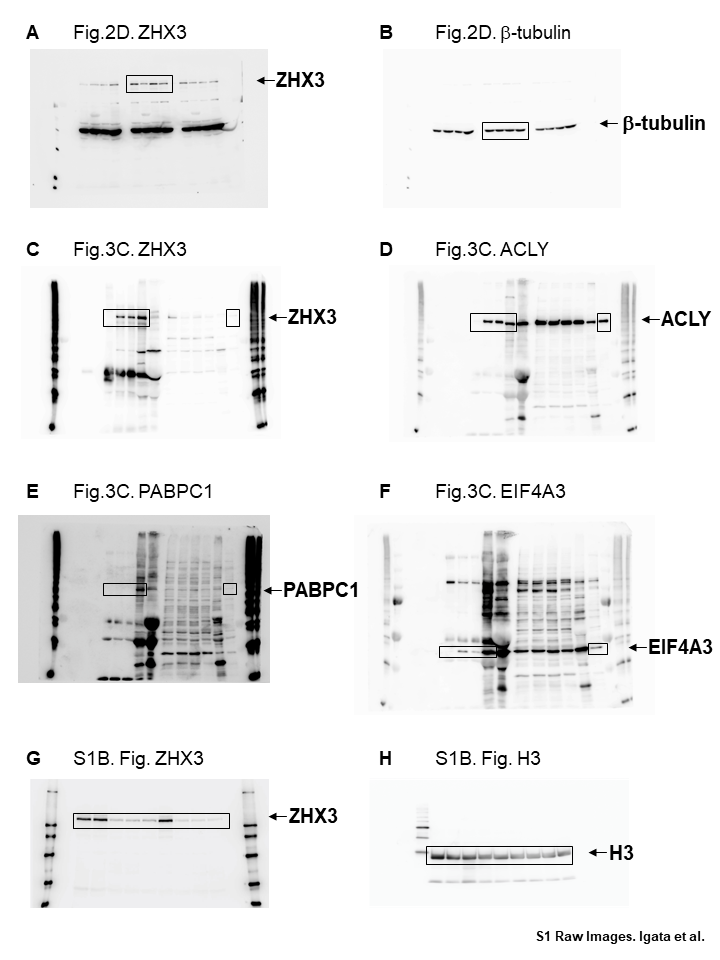

Supplement: S1 Raw images — (TIF) [file pone.0262488.s005.tif]

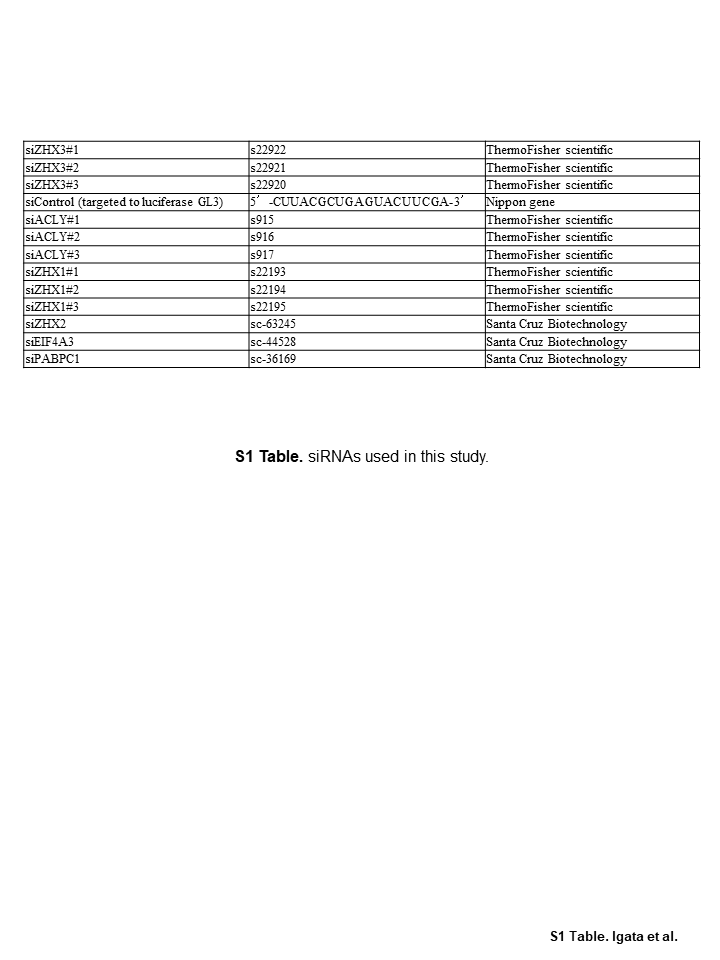

Supplement: S1 Table — (TIF) [file pone.0262488.s006.TIF]

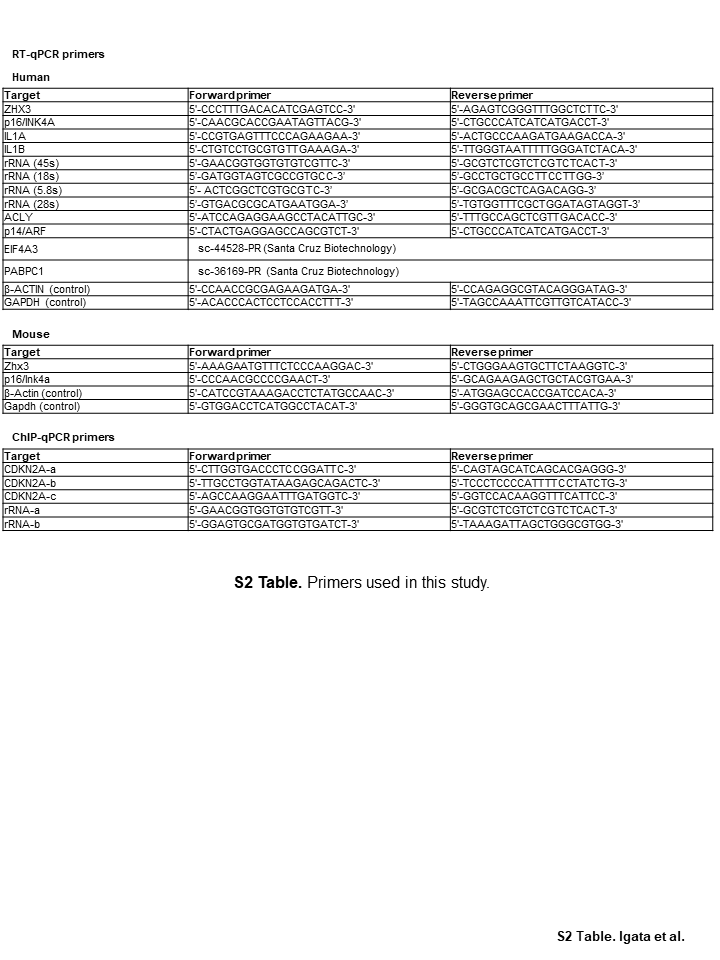

Supplement: S2 Table — (TIF) [file pone.0262488.s007.TIF]

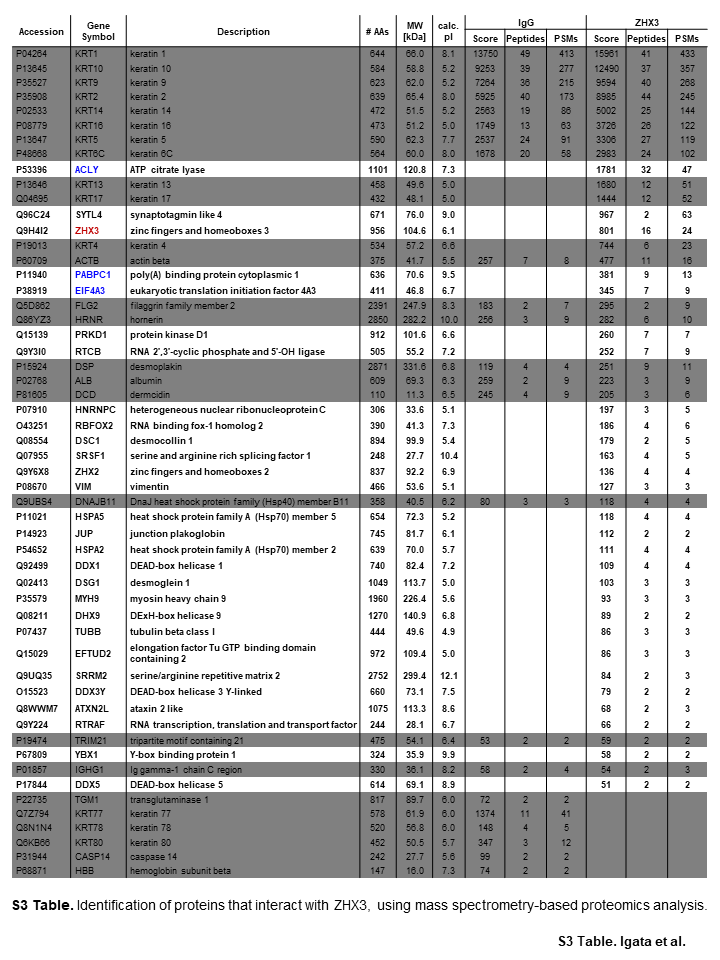

Supplement: S3 Table — Cellular proteins were immunoprecipitated with anti-ZHX3 antibodies, followed by liquid chromatography in tandem with mass spectrometry analyses. The number of Mascot scores, peptide hits and peptide-spectrum matches (PSMs) are included in the list. Non-specific proteins are shaded in gray. (TIF) [file pone.0262488.s008.TIF]
